# Effects of aging on recognition and dominance perception in laughter

**DOI:** 10.3389/fnagi.2026.1816927

**Published:** 2026-06-16

**Authors:** Diana P. Szameitat, Dirk Wildgruber, André J. Szameitat

**Affiliations:** 1 Department of Psychology and Neuroscience, City St George’s, University of London, London, United Kingdom; 2 Department of Psychiatry and Psychotherapy, University of Tübingen, Tübingen, Germany; 3 Centre for Cognitive and Clinical Neuroscience, Department of Psychology, College of Health, Medicine and Life Sciences, Brunel University of London, Uxbridge, United Kingdom

**Keywords:** aging, emotions, joy, laughter, nonverbal communication, schadenfreude, tickling

## Abstract

**Introduction:**

Aging is associated with reduced accuracy in recognizing others’ emotions, an ability that is important for maintaining social connectedness in later life. Laughter is a social signal with multiple functions, as it can facilitate social bonding but also convey negative social meanings, for example when directed at someone. In previous research we have shown that younger adults are able to classify spontaneously emitted joyful, schadenfreude, and tickling laughter above chance level, and that these laughter sounds differ according to the perceived dominance. Given evidence that affect recognition generally declines with age, the present study examined whether comparable age effects emerge in the perception of laughter.

**Methods:**

64 younger adults (mean 25 years, 18–33 years) and 30 older adults (mean age 60 years, 50–77 years) evaluated 117 spontaneously emitted laughter sounds according to the laughter type, i.e., joyful, Schadenfreude, and tickling laughter and according to the perceived sender’s dominance.

**Results:**

Results showed that both age groups classified laughter above chance level. Younger adults showed higher classification rates than older adults for all laughter types, with the largest age effect for Schadenfreude laughter. The dominance ratings showed an age effect only for Schadenfreude, where older adults rated Schadenfreude laughter less dominant than younger adults.

**Discussion:**

Pronounced differences in Schadenfreude perception might be ascribed to difficulties of older adults in perceiving non-literal messages or to cultural differences between age groups.

## Introduction

Aging is accompanied by a range of cognitive and socio-emotional changes that alter how individuals perceive and interpret social situations (Reddy, 2025). A central focus of empirical research is the ability to decode others’ emotions, a skill that is essential for effective social communication. In addition to socio-emotional changes, age-related differences in cognitive resources may also contribute to variability in emotion recognition. Successfully decoding emotional signals often requires the integration of multiple cues under time constraints, placing demands on cognitive processes such as working memory, attention, and processing speed (Gazzaley et al., 2007; Idowu and Szameitat, 2023; Salthouse, 2009). Declines in these cognitive resources may therefore affect how accurately older adults interpret complex social signals, including nonverbal vocalizations such as laughter. Maintaining correct interpretation of the emotional expressions of others’ is particularly important for older adults because impaired social perception might lead to increased difficulties in maintaining social connectedness (Krendl et al., 2022) which in turn can lead to a decline in emotional and physical wellbeing and cognitive functioning (Boss et al., 2015; Shankar et al., 2011) and to an increase in loneliness (Zakahi and Goss, 1995). While a substantial body of research has shown that older adults (OA) exhibit lower accuracy in identifying emotions from faces, speech, and body postures than younger adults (YA) (Ruffman et al., 2008), little is known about the decoding of emotions in nonverbal vocal signals such as screams, laughs, and cries. The present study investigated how younger and older adults interpret laughter, a signal that is particularly important for shaping social connectedness, because laughter can not only facilitate social bonding (Eibl-Eibesfeldt, 1970; Scott, 2013; Szameitat et al., 2022), but it can also carry negative social meanings, for example when used to exclude others from the social group (Provine, 2013; Szameitat et al., 2009). Therefore, the first aim of the current study was to test for age effects in the classification of different laughter types.

It has been shown that older adults are less accurate than younger adults in identifying emotions from emotional facial expressions, emotional speech prosody, body postures and movements, and various combinations of different channels (Baglione et al., 2025a; Gonçalves et al., 2018; Montepare et al., 1999; Ruffman et al., 2008), with effect sizes showing considerable variation across studies (Ruffman et al., 2008). However, previous studies have also shown that changes in recognition abilities in OA cannot simply be described as an overall decline but instead reflect a more complex pattern. Some emotions seem to be more affected by the age decline than others. Rather than being a single unified mechanism, age-related differences in emotional processing have often been discussed in terms of dimensional models of affect, such as valence, arousal, and dominance (Russell and Mehrabian, 1977; Warriner et al., 2013), which may be differentially affected by aging. Within this broader framework, the so-called Positivity Effect, commonly discussed in the context of socioemotional selectivity theory (SST), suggests that older adults tend to prioritize and process positive over negative information (Carstensen et al., 2003) which may influence how social and emotional situations are interpreted. Rather than originating from a single account, this effect has been described across a range of studies on age-related changes in attention and memory (Carstensen et al., 2003; Mather and Carstensen, 2005), and has also been discussed in the context of emotion recognition (Ruffman et al., 2008). SST proposes that older adults focus more on positive information to regulate affect and maintain well-being because they perceive their remaining lifetime as limited and, therefore, increasingly prioritize emotionally meaningful goals (Carstensen et al., 2003). In line with this account, Montepare et al. (1999) reported that the decline in recognition accuracy in OA when interpreting emotions from short video segments of body movements is especially pronounced for negative emotions such as anger and sadness, while neutral and happy expressions were not affected. Consistent with this pattern, several studies observed that OA showed a pronounced reduction in accuracy when decoding negative emotional facial expressions and speech prosody, such as anger, sadness, and fear, but less so for positive emotions, such as happiness and surprise (Hayes et al., 2020; Mill et al., 2009; Nasrollahi et al., 2022; Ruffman et al., 2008). However, there are also many studies which have been unable to replicate this pattern (Demenescu et al., 2014; Fölster et al., 2015; Lima et al., 2014; Orgeta and Phillips, 2007) and it has been suggested that the pattern is more nuanced than initially assumed (Laulan and Rimmele, 2024). Overall, prior research suggests that age-related declines in emotion recognition vary across emotions, though the exact pattern of decline remains unclear.

While age effects in the perception of emotions in facial expressions and speech prosody have been studied extensively, less is known about the ability of OA to correctly interpret nonverbal vocal signals, such as screams, sobs, and laughs. In previous studies, OA and YA classified diverse positive and negative nonverbal vocalizations according to the emotional category, e.g., laughter, screams, or sobs (Amorim et al., 2021; Cortes et al., 2021; Lima et al., 2014), and all studies showed age-related decline in the accuracy of assigning the vocalization to the intended emotion. Lima et al. (2014) found that the effect was consistent for positive as well as negative emotions and independent of hearing abilities and cognitive abilities. Amorim et al. (2021) reported that between the ages of 23 and 60, decoding abilities declined for all emotions except for fear and surprise, with the strongest declines observed for pleasure and sadness. Therefore, results for nonverbal vocalizations do not support the existence of a positivity effect. In addition, despite growing evidence into the perception of diverse nonverbal signals, little is known about how older and younger adults perceive different expressions within a single nonverbal vocalization, such as laughter. To shed light on this, the present study investigated how older and younger adults perceive different expressions of a single nonverbal vocalization, namely laughter. Laughter will be taken from a corpus of spontaneously emitted laughter sounds that were uttered in various social situations, such as individuals tickling each other or watching funny video clips together (Szameitat et al., 2022).

Aside from the ability to classify emotions into categories, it is also important to examine age effects in terms of emotional dimensions of affect (Russell and Mehrabian, 1977; Warriner et al., 2013). One such dimension is dominance, which reflects the extent to which a signal conveys social power, hierarchy, and interpersonal intent, and is therefore a core component of social evaluation (Russell and Mehrabian, 1977; Scherer, 2003). Laughter, in particular, is not only an expression of affect but also a social signal that helps regulate group dynamics and negotiate social relationships (Dezecache and Dunbar, 2012), for example by conveying affiliation, superiority, or exclusion (Oveis et al., 2016; Scott et al., 2014; Szameitat et al., 2022; Wood et al., 2017). Assessing how dominant a laugh is perceived to be may therefore provide important insight into how individuals interpret others’ social intentions. Misinterpretations in this domain could contribute to poorer judgments about others’ motives. Such misinterpretations may have real-world consequences especially for older adults which might leave them more vulnerable to deception and fraud (Shao et al., 2019). Regarding the dominance dimension, there is only limited evidence of age-related effects on the perception of vocal signals. McGettigan and Lavan (2023), who investigated the influence of listener age on the perception of various personality traits, found no differences between younger and older participants when judging dominance in audio recordings of sustained vowels. However, von der Rosenthal- Pütten et al. (2019), who examined how younger and older adults interpreted communications produced by virtual agents, found that older adults judged the virtual agent to be more submissive than did younger adults. In line with this, Fairfield et al. (2017), who investigated the perception of arousal, valence, and dominance in Italian words, reported that older adults assigned lower dominance ratings to words that younger adults had rated as moderately dominant. Overall, prior research suggests that older adults may perceive vocal signals as less dominant than younger adults. However, the evidence is inconsistent and particularly limited with respect to laughter. Therefore, the second aim of this study was to test for age effects in the dominance perception of different laughter types.

Laughter is a nonverbal vocalization that is uttered in a wide variety of contexts. It has been described as an expression of mere joy and happiness (Darwin, 1872) but is also uttered when experiencing emotions other than joy (Poyatos, 1980). It is expressed in various different situational contexts, such as when being tickled (Kamiloğlu et al., 2024), in social interactions (Vettin and Todt, 2004), when sharing humour (Ruch et al., 2001), or as a response to another person’s laughter (Smoski and Bachorowski, 2003). In these contexts, laughter is typically associated with positive affect and functions to promote the strengthening of social bonds and group cohesion (Dezecache and Dunbar, 2012; Eibl-Eibesfeldt, 1970; Scott, 2013). However, laughter can also carry negative social meanings, for example when used to belittle or mock others, to express hierarchy or social dominance, or to exclude others from the social group (Klages and Wirth, 2014), for example when the person who laughs expresses schadenfreude (i.e., laughing at the misfortune of someone), derision or triumph (Provine, 2013; Szameitat et al., 2009). Schadenfreude is not merely amusement at another person’s misfortune but reflects a socially and morally complex emotional response. Smith et al. (2009) argue that schadenfreude is shaped by multiple social-cognitive processes, including social comparison, envy, deservingness judgments, and moral evaluation. Specifically, they propose that schadenfreude is more likely to arise when another person’s misfortune is perceived as restoring justice or correcting an unfair advantage. Supporting the role of deservingness, van Dijk et al. (2005) demonstrated experimentally that individuals experience stronger schadenfreude when the target’s negative outcome is judged as deserved rather than undeserved. Likewise, Feather and Sherman (2002) showed that schadenfreude is particularly pronounced when a previously successful or high-status target experiences failure, especially when that prior success is perceived as undeserved. This finding highlights the importance of social comparison processes and envy, as the misfortune of someone perceived as more successful can make people feel better about themselves and may elicit pleasure. Together, these findings suggest that schadenfreude depends on evaluative judgments about fairness, status relations, and interpersonal comparison, linking it closely to broader processes of moral and social evaluation that are likely shaped, at least in part, by culturally shared social norms.

In a recent study, we have shown that spontaneously emitted laughs can be classified according to the emotional state of the person who laughs with higher than chance accuracy and that different laughter types, i.e., tickling laughter (i.e., laughter emitted when being tickled), joyful laughter, and schadenfreude laughter, show distinct patterns when rated according to various emotional dimensions, when evaluated by younger participants (mean age 23.3 years) (Szameitat et al., 2022). However, it remains unclear whether older adults are equally able to distinguish between these forms of laughter and whether they rate laughter on emotional dimensions similar to younger adults.

To address these gaps, we conducted two experiments aimed at examining age effects in the perception and interpretation of laughter. In Experiment 1, younger (YA; 18–33 years, mean 25 years) and older adults (OA; 50–77 years, mean 60 years) classified laughter into categories, i.e., joyful, schadenfreude, and tickling laughter. Based on existing findings from emotional facial and vocal expressions, we hypothesized that older adults would exhibit reduced classification accuracy compared to younger participants. In Experiment 2, participants interpreted laughter in terms of the sender’s intention, specifically focusing on the perceived dominance. We hypothesized that older adults perceive laughter as less dominant overall compared with younger adults. Together, these studies aim to contribute to an understanding of how age affects the decoding of complex social signals conveyed through nonverbal vocalizations.

## Methods

### Participants

Overall, 94 German participants were recruited. The younger adult [YA] group consisted of 64 participants (32 female, mean age 25 years, SD 4 years, min 18, max 33 years). The older adult [OA] group consisted of 30 participants (15 female, mean age 60 years, SD 4 years, min 50, max 77 years). Sixty-four younger participants were tested because data were part of another study not published yet. A power analysis (GPower 3.1.9.7) for an independent-samples t-test directly comparing YA with OA (assuming three t-tests, one per laughter type) with an allocation ratio of 2:1 (younger: older aged participants) with two-tailed alpha = 0.05 and an assumed effect size of 0.65 (Szameitat and Szameitat, 2024) indicated that 57 younger and 29 older participants would be required for a power of 0.80.

Participants were included if they were at least 18 years of age, native German speakers, and reported normal hearing. Participants were excluded if they reported hearing impairments, a history of neurological disorders, cognitive impairments, or if they reported being on the autism spectrum. In addition, after data collection, participants were excluded if they reported that they were not able to concentrate until the end of the experiment.

As assessed via initial screening criteria and a self-report questionnaire, none of the participants had impaired hearing abilities or a history of neurological disorders. Before the experiment, participants were asked if they were familiar with the terms “Freude,” (joy) “Schadenfreude” (German term for “laughing at another’s misfortune”), and “Kitzeln” (tickling) and all participants confirmed that they were. They were then asked to provide an example situation for each scenario, and all participants could name an example that matched.

In YA, one participant was excluded from both tasks due to an Autistic Spectrum Disorder diagnosis. An additional three participants were excluded from the classification task due to technical problems. The final YA sample consisted of 60 participants for the laughter classification task (30 females, 30 males) and 63 for the dominance task (32 females, 31 males). In OA, two participants were excluded from the Dominance analysis because both participants reported difficulties concentrating during the task, which was always presented after the laughter classification task. To not put any mental strain on the participants, the dominance task was stopped before completion. The final OA sample consisted of 30 participants for the laughter classification task (15 females, 15 males), and 28 for the dominance task (15 females, 13 males).

All studies in this manuscript were approved by the ethical review board of the Medical Faculty of the University of Tübingen, Germany (225/2010BO1). Participants were recruited through circular emails and flyers posted on student notice boards. In addition, for the recruitment of OA participants opportunistic sampling was used, involving an article in the local newspaper. All participants received 10 Euro per hour for their participation.

### Stimulus material

A set of 117 spontaneous laughter stimuli generated by 39 German speakers in a previous study (Szameitat et al., 2022) was used. For stimulus generation, groups of friends (speakers/senders) were invited to do various activities together. They tickled each other (Ticking laughter) and watched funny video clips together. Notably, participants frequently laughed in response to the videos and the tickling. Various video clips were chosen to elicit different emotional responses, i.e., some depicted positive funny situations (e.g., a newsreader misreading and laughing at herself, a baby laughing funnily, etc), while other video clips were meaner in nature (e.g., clips of nasty pranks, such as letting a friend step into mouse traps). After watching the video clips, participants answered a questionnaire in which they were asked what emotions they felt during each individual video clip. Joyful and schadenfreude laughter were defined as laughter expressed while the participant experienced joy (Joyful laughter) or schadenfreude (Schadenfreude laughter, i.e., laughing at another’s misfortune). This approach resulted in a set of 381 laughter stimuli.

In a second step, each laughter sound was classified according to its underlying emotion by native German participants who did not take part in the laughter recordings and who served as independent naïve listeners. Data were collected in three separate experiments (42–66 participants per experiment, total *N* = 156, 85 men, 74 women, mean age = 23.3 years, Szameitat et al., 2022). Based on those results, a sub-set of 117 stimuli was generated, in which each individual laughter stimulus had a correct classification rate of at least 50%. For more details on stimulus generation and laughter classification, please refer to Szameitat et al. (2022).

The resulting stimulus set consisted of 117 laughter sequences (46 joy, 42 tickling, 29 schadenfreude, 1–4 per laughter type and sender, 19 male and 20 female senders, average age 23.9 years old, s.d. 2.2 years) with an average classification rate of 56.3% (tickling 52.8%, joy 54.5%, schadenfreude 63.4%). Please note that it is not possible to directly compare the results from Szameitat et al. (2022) with the current study due to some methodological differences (e.g., Szameitat et al. used four possible answer categories instead of three).

### Procedure

The study was conducted in standard laboratories at the University of Tübingen, Germany. Before the experiment, participants gave informed consent, received instructions, and practiced with 12 laughter sequences that were not part of the final stimulus set of 117 laughter sequences.

The study was programmed in Presentation (Neurobehavioral Systems, www.neurobs.com) and both experiments (Experiment 1: laughter classification, Experiment 2: dominance rating) included the same 117 stimuli that were split into four blocks of 29–30 stimuli for each task, whereby stimulus order was randomised across participants.

For experiment 1, in which participants were asked to classify laughter according to the laughter type, participants listened to the whole laughter sequence first and then had to indicate whether the laugh sounded like joyful laughter, schadenfreude laughter (i.e., laughing at the misfortune of somebody else), or laughter provoked by tickling. To provide the answer, participants clicked on one of three answer boxes displayed on the screen using a computer mouse. They could listen to each laughter sequence as often as they liked before they gave their answer, after which the next laughter sequence was played. There was no time pressure during the experiment and participants had the option to take a break between blocks. The average duration of the experiment was 17 min. Experiment 1 was always presented first.

For experiment 2, participants were asked: “How dominant does the laughter sound to you? This means whether the person who laughs tries to domineer you or is submissive”. Participants were asked to click on one of four answer boxes displayed on the screen, using a 4-point Likert scale ranging from “−−” (the person is very submissive), “−” (the person is submissive), “+” (the person is dominant), to “++” (the person is very dominant). The experiment was delivered in the same way as Experiment 1.

### Statistical analysis

For laughter classification, one outlier was removed for the younger adult group for schadenfreude laughter. For dominance ratings, two outliers were excluded for the younger adult group: one for schadenfreude laughter and one for joyful laughter. There were no outliers for the older adults group. Outliers were defined as individual means more than three standard deviations from the group means.

For all statistical tests of Experiment 1, Wagner’s (1993) unbiased hit rate for correct classification (H_u_) and Wagner’s proportion correct (p_c_) was calculated for each individual participant and laughter type. By comparing H_u_ with p_c_ this method allows to account for uneven stimulus distributions, false alarms, and response biases (Wagner, 1993). For convenience of the reader, hit rates are reported in the main text, tables, and figures.

For experiment 2, response frequencies were multiplied with a factor (1.5 for + +, 0.5 for +, −0.5 for –, and −1.5 for − −) and summed. To render a scale ranging from −100 to + 100, this sum was divided by the highest possible sum (which depended on the number of stimuli in that category) and multiplied by 100.

## Results

### Experiment 1: laughter classification

#### Descriptive statistics

**Figure 1:**
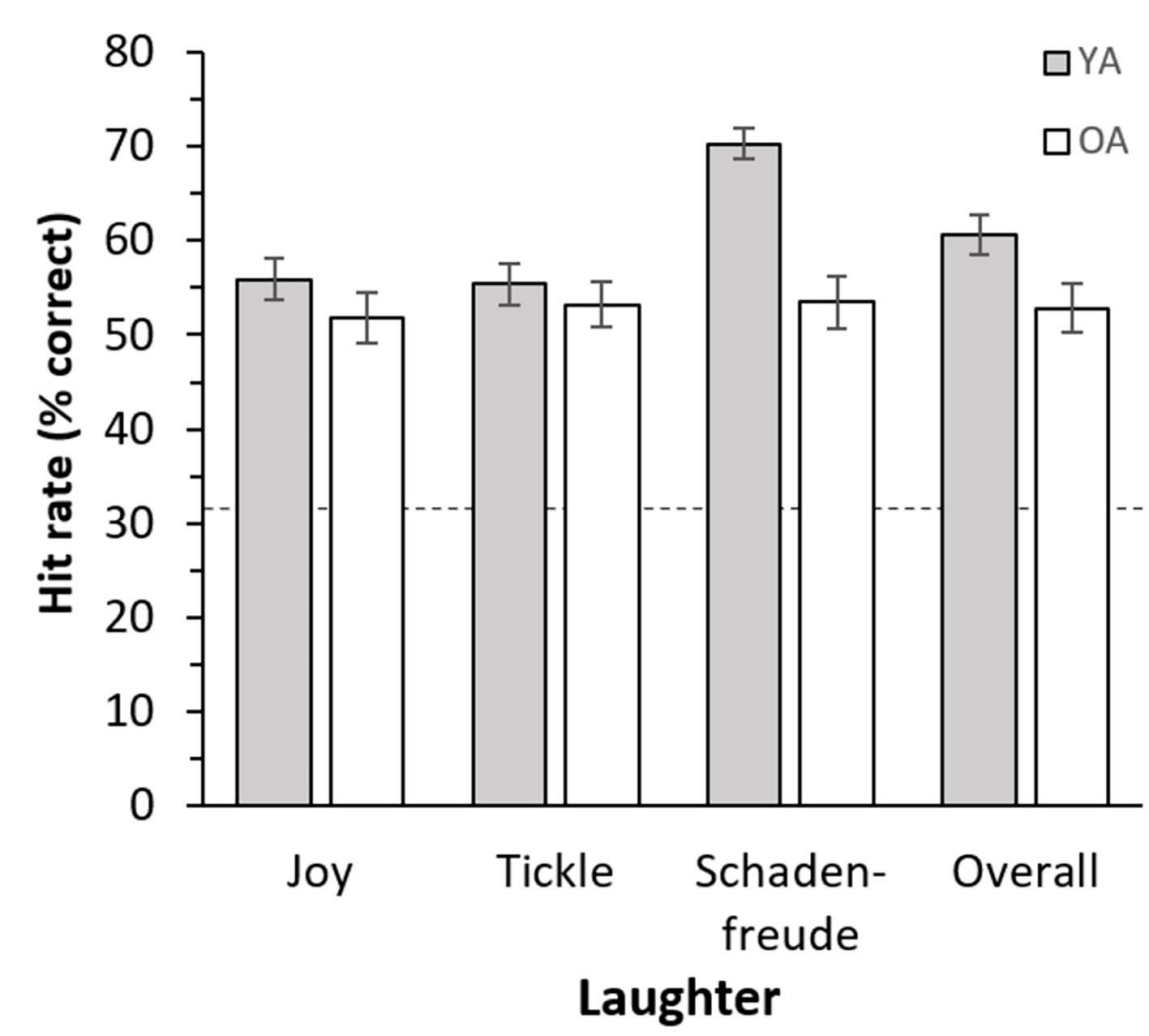
Classification rates (percent correct) for the three laughter types, separated by age groups. Error bars denote standard error of the mean (SEM). Dotted line shows 33% guessing probability (chance level). Younger adults (YA), older adults (OA). Overall shows the average across laughter types.

Classification rates were on average 60.7% (chance level 33%) for YA and 52.8% for OA with the highest recognition rate (70.6%) for schadenfreude laughter in YA, and the lowest recognition rate (51.8%) for joyful laughter in OA (Table 1; Figure 1).

**Table 1:** Correct classification rates for the three laughter types for separate age groups.

Group	Joy	Schadenfreude	Tickling	Overall
Younger Adults (YA)	55.9% (2.2)	70.3% (1.7)	55.4% (2.2)	60.7% (2.1)
Older Adults (OA)	51.8% (2.6)	53.5% (2.8)	53.2% (2.4)	52.8% (2.6)

Shown are the hit rates (% correct) and the standard error of the mean (SEM) in parentheses.

#### Can laughter be recognised within each age group?

All of the following statistical tests were based on Wagner’s (1993) unbiased hit rate for correct classification (H_u_) and on Wagner’s proportion correct (p_c_), individually calculated for each participant and laughter type. To test whether both age groups were able to recognise the three laughter types above chance level we calculated one sample t-tests, separate for each age group. In detail, correct classification was tested calculating relative differences between H_u_ and p_c_ (i.e., H_u_ minus p_c,_ [H_u_-p_c_]). If H_u_ and p_c_ were identical (i.e., the difference is 0) participants would not be able to recognise the laughter type above chance level.

Both participant groups classified all three laughter types well above chance level (one sample *t*-tests H_u_-p_c_ vs. 0; Bonferroni corrected for 6 comparisons; all *p* < 0.001; YA: Joy: *t*(59) = 14.2, Cohen’s *d* = 1.83, Schadenfreude: *t*(58) = 24.2, Cohen’s *d* = 3.15, Tickling: *t*(59) = 18.8, Cohen’s *d* = 2.43; OA: Joy: *t*(29) = 9.02, Cohen’s *d* = 1.65, Schadenfreude: *t*(29) = 9.31, Cohen’s *d* = 1.70, Tickling: *t*(29) = 17.58, Cohen’s *d* = 3.21). This shows that both participant groups were able to classify laughter according to the laughter type significantly above chance level.

#### Do the recognition rates differ between age groups?

We hypothesized that OA would have lower recognition rates in general. To test whether participant age or laughter type influenced laughter perception, we performed a 2 × 3 mixed ANOVA with the between-subject factor Age (YA, OA) and the within subject factor Laughter Type (Joy, Schadenfreude, Tickling), and with (H_u_ minus p_c_) as the dependent variable. Across laughter types, OA showed significantly lower recognition rates than YA (main effect Age, *F*(1, 87) = 21.5, *p* < 0.001, partial *η^2^* = 0.20). In addition, across age groups, the recognition rates differed significantly between laughter types (main effect Laughter Type, *F*(2, 174) = 51.84, *p* < 0.001, partial *η^2^* = 0.37). The interaction between Age and Laughter Type was highly significant as well (*F*(2, 174) = 7.70, *p* < 0.001, partial *η^2^* = 0.08).

To scrutinise the main effect of Age in more detail, we tested whether younger adults performed better than older adults for each individual laughter type by calculating independent samples t-tests. These analyses revealed that YA showed a significantly higher classification rate as compared to OA when the laughter types were analysed separately (independent samples *t*-test of H_u_−p_c_ scores of the YA group vs. H_u_−p_c_ scores of the OA group: Joy: *t*(88) = 2.15, *p* = 0.034, *p*_Bonferroni_ = 0.102, Cohen’s *d* = 0.48, Schadenfreude: *t*(87) = 6.64, *p* < 0.001 *p*_Bonferroni_ <0.001, Cohen’s *d* = 1.49, Tickling: *t*(88) = 2.71, *p* = 0.008 *p*_Bonferroni_ <0.024, Cohen’s *d* = 0.61). This shows that younger adults, as compared to older adults, showed a higher classification rate for every laughter type (see Figure 1 and Table 1 for hit rates for each individual laughter type and age group).

As reported above, the 2 × 3 mixed ANOVA revealed a significant interaction between Age and Laughter Type. To further examine this interaction, we conducted three follow-up 2 × 2 mixed ANOVAs, each including the between-subjects factor Age (YA vs. OA) and the within-subjects factor Laughter Type. Three separate analyses were performed, one for each pairwise comparison of laughter types (Schadenfreude vs. Joy, Schadenfreude vs. Tickling, Joy vs. Tickling, dependent variable always H_u_-p_c_).

These analyses showed that the age effect in recognition accuracy was significantly larger for schadenfreude laughter than for joyful laughter (2 × 2 ANOVA with Age (OA vs. YA) and Laughter Type (Schadenfreude vs. Joy); interaction Age x Laughter Type: *F*(1,87) = 14.9, *p* < 0.001, partial *η^2^* = 0.15) and tickling laughter (2 × 2 ANOVA with Age (OA vs. YA) and Laughter Type (Schadenfreude vs. Tickling); interaction term: *F*(1,87) = 6.91, *p* = 0.010, partial *η^2^* = 0.07). In contrast, the interaction Age x Laughter Type was not significant for joyful and tickling laughter (2 × 2 ANOVA with Age (OA vs. YA) and Laughter Type (Joy vs. Tickling); interaction term: *F*(1,88) = 1.16, *p* = 0.285, partial *η^2^* = 0.013). Thus, the age-related decline in recognition performance was most pronounced for schadenfreude laughter (Figure 1).

The three 2 × 2 ANOVAs also allowed for a more detailed examination of the main effects. First, the main effect of Age was significant in all analyses (Schadenfreude vs. Joy: *F*(1,87) = 27.7, *p* < 0.001, partial *η^2^* = 0.24, Schadenfreude vs. Tickling: *F*(1,87) = 27.2, *p* < 0.001, partial *η^2^* = 0.24, Joy vs. Tickling *F*(1,88) = 7.81, *p* = 0.0064, partial *η^2^* = 0.08), confirming the results of the initial 2 × 3 ANOVA. Second, the main effect of Laughter type was significant in all analyses (Schadenfreude vs. Joy: *F*(1,87) = 25.5, *p* < 0.001, partial *η^2^* = 0.23, Schadenfreude vs. Tickling: *F*(1,87) = 24.38, *p* < 0.001, partial *η^2^* = 0.22, Joy vs. Tickling *F*(1,88) = 115.28, *p* < 0.001, partial *η^2^* = 0.57), confirming the results of the initial 2 × 3 ANOVA. Follow-up tests showed that recognition rates were highest for schadenfreude laughter, followed by tickling laughter, with joyful laughter showing the lowest recognition rates (paired sample t-tests H_u-pc_; Bonferroni corrected for 3 comparisons; pairwise comparisons for all three laughter types, all p < 0.001; all *t*(88) > 4.18, all Cohen’s *d* > 0.44, average percent correct across OA and YA: Schadenfreude: 61.9%, Tickling: 54.3%, Joy: 53.9%).

#### Confusion matrices

To further examine the response patterns, confusion matrices were calculated separately for younger adults (YA; Table 2) and older adults (OA; Table 3). Overall, YA and OA showed highly similar response distributions for Joy and Tickle stimuli. The most notable age-related difference emerged for Schadenfreude stimuli: OA selected Schadenfreude (the correct response) 17.03% less often than YA (53.60% vs. 70.63%). Instead, these responses were redistributed to Joy (+9.94%) and Tickle (+7.10%).

**Table 2:** Confusion matrix for the younger adults (YA).

		Response		
		Joy	Schadenfreude	Tickle
STIMULUS	JOY	**55.94%**	30.51%	13.55%
	SCHADENFREUDE	20.57%	**70.63%**	8.79%
	TICKLE	31.15%	13.45%	**55.40%**

Shown are how many percent of the stimuli of each laughter type are classified as Joy, Schadenfreude, and Tickle, respectively. Bold diagonal corresponds to correct classification.

**Table 3:** Confusion matrix for the older adults (OA).

		Response		
		Joy	Schadenfreude	Tickle
STIMULUS	JOY	**51.86%**	31.61%	16.53%
	SCHADENFREUDE	30.51%	**53.60%**	15.89%
	TICKLE	28.08%	18.80%	**53.12%**

Shown are how many percent of the stimuli of each laughter type are classified as Joy, Schadenfreude, and Tickle, respectively. Bold diagonal corresponds to correct classification.

Importantly, however, the relative pattern of misclassification was comparable across groups. Among YA, Joy was selected for Schadenfreude stimuli approximately twice as often as Tickle (20.57% vs. 8.79%, ratio = 2.3:1). A similar pattern was observed in OA, for whom Joy was also chosen roughly twice as often as Tickle (30.51% vs. 15.89%, ratio = 1.9:1). Thus, although OA were less likely than YA to correctly identify Schadenfreude stimuli, their overall misclassification pattern closely resembled that of YA, suggesting a quantitative reduction in recognition accuracy rather than a qualitatively different response strategy.

A possible explanation for the lower recognition accuracy observed in OA is that they may generally distribute their responses differently across the available response categories, regardless of laughter type. To test this possibility, we calculated the average number of responses assigned to each category separately for YA and OA. As shown in Table 4, response distributions were highly similar across groups. OA gave slightly fewer Schadenfreude responses (on average 2.19 fewer responses out of 117), which were correspondingly redistributed to Tickle. However, this difference was not statistically significant, and the overall response distributions did not differ between YA and OA (χ^2^(2) = 0.304). These findings indicate that a general response bias cannot account for the lower recognition accuracy for Schadenfreude observed in OA.

**Table 4:** Distribution of the average responses for the different response categories (Joy, Schadenfreude, Tickle), averaged across the laughter types (stimuli).

Group	Joy	Schadenfreude	Tickle	SUM
Younger adults (YA)	44.78	40.16	32.05	117
Older adults (OA)	44.52	37.97	34.51	117

Numbers given in average responses (the experiment had 117 trials).

### Experiment 2: dominance rating

#### Descriptive statistics

Average dominance ratings on a scale of −100 to +100 were on average +10.12 for YA and +10.35 for OA with the highest values (+32.63) for schadenfreude laughter in YA, and the lowest values (−3.75) for joyful laughter in YA (Table 5).

**Table 5:** Mean dominance ratings for the three laughter types for separate age groups.

Group	Joy	Schadenfreude	Tickling	Overall
Younger adults (YA)	2.48 (2.2)	32.63 (2.4)	−3.75 (3.2)	10.12 (2.6)
Older adults (OA)	4.73 (2.7)	24.2 (2.8)	2.14 (3.9)	10.35 (3.2)

Shown are the mean and the standard error of the mean (SEM) in parentheses, on a scale from −100 (maximal submissive) to +100 (maximal dominant).

#### Do dominance ratings differ between age groups?

To test whether dominance ratings differed between younger and older adults, we conducted a 2 × 3 mixed ANOVA with the between-subject factor Age (YA, OA) and the within-subject factor Laughter type (Joy, Schadenfreude, Tickling). Dominance ratings were transformed to a scale ranging from −100 (maximal submissiveness) to +100 (maximal dominance), with 0 indicating a neutral dominance rating.

**Figure 2:**
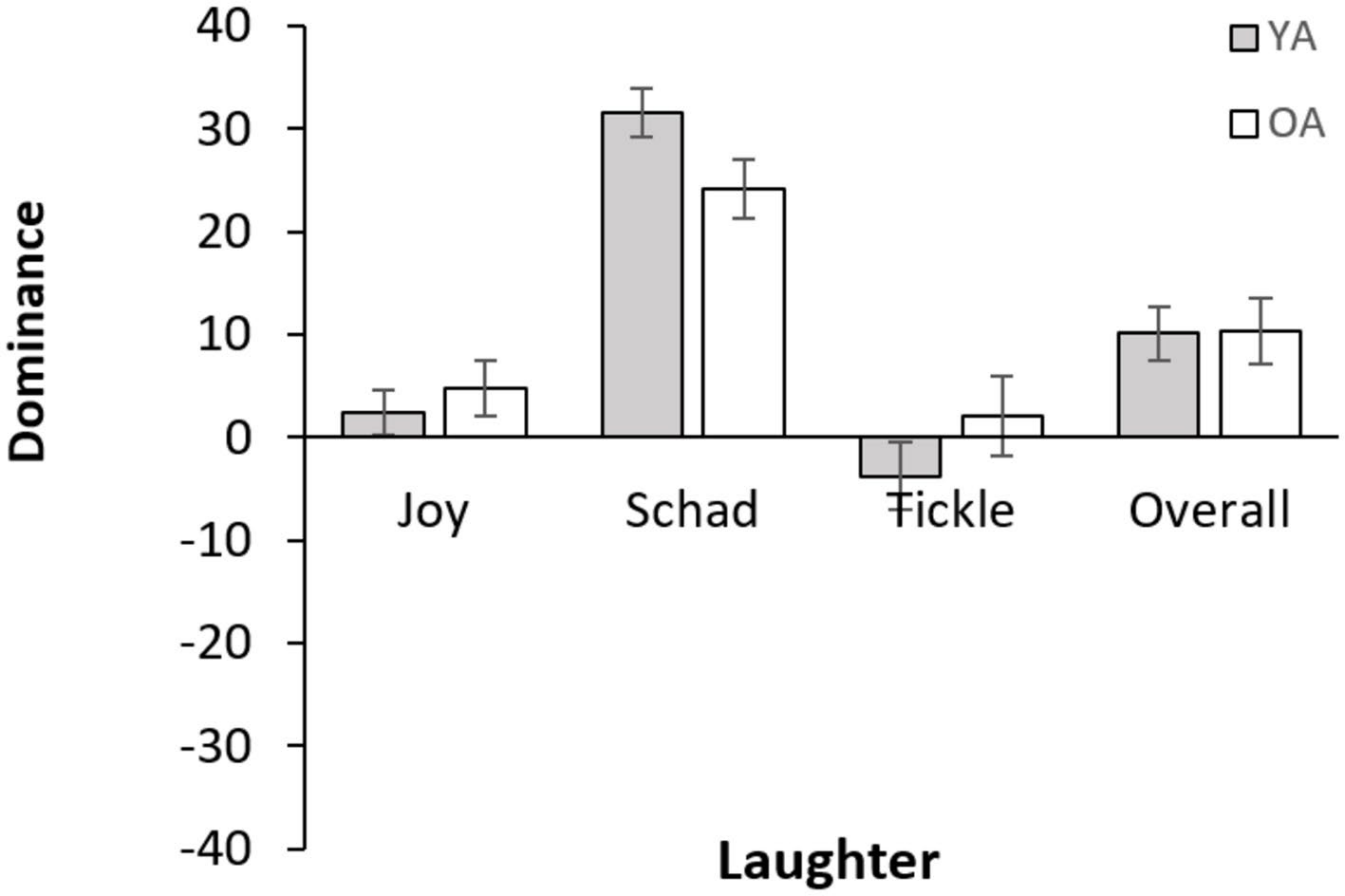
Dominance rating per laughter type, split by age group. Error bars denote standard error of the mean (SEM). Younger adults (YA), older adults (OA). Overall shows the average across laughter types.

Dominance ratings did not differ between age groups (Figure 2, main effect Age, *F*(1, 87) = 0.04, *p* = 0.843, partial *η^2^* < 0.01). However, across age groups, dominance ratings differed significantly between laughter types (main effect Laughter Type, *F*(2, 174) = 69.51, *p* < 0.001, partial *η^2^* = 0.44). The interaction between Age and Laughter Type was significant (*F*(2, 174) = 3.34, *p* = 0.038, partial *η^2^* = 0.04), showing that the effect of Age differed significantly between Laughter Types.

To explore the interaction between Age and Laughter Type in the 2 × 3 mixed ANOVA in more detail, we additionally performed three follow-up 2 × 2 mixed ANOVAs. Each analysis included the between-subjects factor Age (YA vs. OA) and the within-subjects factor Laughter Type, with three separate analyses, one for each pairwise comparison of laughter types (Schadenfreude vs. Joy, Schadenfreude vs. Tickling, Joy vs. Tickling). The Age x Laughter Type interaction was significant for schadenfreude vs. joyful laughter (*F*(1,87) = 8.04, *p* = 0.006, partial *η^2^* = 0.09), and for schadenfreude versus tickling laughter (*F*(1,88) = 4.35, *p* = 0.040, partial *η^2^* = 0.05), but was not significant for joyful versus tickling laughter (*F*(1,88) = 0.23, *p* = 0.634, partial *η^2^* < 0.01). The main effect of Age on dominance perception was not significant across all comparisons (Schadenfreude vs. Joy: *F*(1,87) = 1.05, *p* = 0.309, partial *η^2^* = 0.01, Schadenfreude vs. tickling: *F*(1,88) = 0.04, *p* = 0.836, partial *η^2^* = <0.01, Joy vs. tickling *F*(1,88) = 0.90, *p* = 0.344, partial *η^2^* = 0.01). The significant interactions show that the effect of Age differed significantly depending on the Laughter Type.

To further understand the pattern of the above interactions, we calculated three independent-samples t-tests comparing YA with OA, separate for each Laughter Type. These t-tests revealed that the dominance ratings did not differ significantly between YA and OA for Joy and Tickling laughter (Joy: *t*(88) = −0.602, *p* = 0.549, Cohen’s *d* = 0.14; Tickling: *t*(88) = −1.057, *p* = 0.293, Cohen’s *d* = 0.24). However, the difference between YA and OA very closely approached significance for Schadenfreude (*t*(88) = 1.862, *p* = 0.066, Cohen’s *d* = 0.42). These t-test results suggest that the significant interactions reported above are mostly explained by OA having rated only Schadenfreude as less dominant than YA, while the ratings did not differ for Joy and Tickling (see Table 5; Figure 2). In summary, we take the significant interaction terms of the ANOVAs and the near-significant age effect in Schadenfreude as evidence that the OA group perceived specifically Schadenfreude as less dominant than the YA group.

To test whether younger adults and older adults perceived individual laughter types as significantly dominant or submissive, we calculated one-sample *t*-tests versus an average neutral dominance rating of zero for each age group and laughter type separately. These analyses revealed that schadenfreude laughter was perceived as dominant by younger adults (Table 5; Figure 2, *t*(61) = 13.39, *p*_Bonferroni_ <0.001, Cohen’s *d* = 1.70) and older adults (*M* = 24.17, *SEM* 2.84, *t*(27) = 8.51, *p*_Bonferroni_ <0.001, Cohen’s *d* = 1.61), whereas joy and tickling laughter were neither perceived as dominant nor submissive (YA: Joy: *t*(61) = 1.13, *p*_uncorrected_ = 0.263, Cohen’s *d* = 0.14, YA: Tickling: *t*(62) = 1.16, *p*_uncorrected_ = 0.251, Cohen’s *d* = 0.15, OA: Joy: *t*(27) = 1.75, *p*_uncorrected_ = 0.092, Cohen’s *d* = 0.33, OA: Tickling: *t*(27) = 0.53, *p*_uncorrected_ = 0.604, Cohen’s *d* = 0.10).

## Discussion

### Summary of results

The aim of the present study was to test whether younger adults (YA) and older adults (OA) show differences in the classification and dominance perception in laughter. While both participant groups classified laughter sounds above chance level, OA showed a lower recognition ability for all laughter types. Age differences were most pronounced for the classification of schadenfreude laughter. Regarding the perception of dominance, OA perceived specifically Schadenfreude laughter as less dominant than YA, while YA and OA perceived Joy and Tickling as similarly dominant.

### Overall ability to classify laughter across age groups

The present study extends previous literature on processing of nonverbal vocal emotional expressions by demonstrating that laughter encodes nuanced social and emotional information that both, younger and older listeners can decode with above-chance accuracy. A number of studies have previously shown that nonverbal emotional vocalizations can be reliably classified according to the emotion above chance level when diverse types of vocalizations are presented, such as sighs, groans, laughs, and screams (Amorim et al., 2021; Cortes et al., 2021; Lima et al., 2014). The present study extends previous findings by demonstrating that different emotions expressed within a single type of vocalization, i.e., laughter, are recognisable across age groups. Moreover, to our knowledge, this is the first study to examine age effects in the processing of spontaneously emitted nonverbal vocalizations, as opposed to volitionally produced nonverbal vocalizations elicited on command (Amorim et al., 2021; Cortes et al., 2021; Lima et al., 2014).

A recent study on affective prosody comprehension suggests that older adults may experience greater difficulty with low-arousal emotions (Baglione et al., 2025b). Applying this account to the present findings, one might expect reduced classification accuracy for laughter types associated with lower arousal. However, previous work with the same stimulus set (Szameitat et al., 2022) showed that both joyful and schadenfreude laughter are characterized by relatively neutral arousal levels, whereas tickling laughter is associated with high arousal. If age-related differences were primarily driven by arousal, strong age-effects would be expected for the low-arousal joyful and schadenfreude laughter, and smaller effects for the high-arousal tickling laughter. In contrast, the present results showed the strongest age-effect in the low-arousal schadenfreude, and comparatively smaller age-effects in the high-arousal tickling and low-arousal joy laughter. This pattern suggests that differences in arousal alone are unlikely to account for the observed age-related effects and instead points to additional factors specific to schadenfreude laughter, such as its more complex social meaning.

### General age effects in laughter classification

In line with our hypothesis, we found an age effect in laughter classification with older adults showing reduced recognition accuracy across all laughter types, i.e., joyful, tickling, and schadenfreude laughter. This finding is in line with extensive research on classification of emotional facial expression, emotional speech prosody, and body postures and movements (Baglione et al., 2025a; Gonçalves et al., 2018; Montepare et al., 1999; Ruffman et al., 2008) as well as with previous evidence on classification of nonverbal emotional vocalizations (Amorim et al., 2021; Cortes et al., 2021; Lima et al., 2014).

Age-related decline in laughter recognition might be linked to a general age-related decline in cognitive resources, including working memory, processing speed, attention, and executive functioning (Gazzaley et al., 2007; Idowu and Szameitat, 2023; Salthouse, 2009). Emotion recognition tasks require rapid integration of multiple, subtle cues (Choo et al., 2025). Thus, reduced processing efficiency may hinder OA’s ability to decode laughter. However, while it has been shown that OA’s poorer classification of emotional prosody correlates with a decline in working memory and verbal intelligence (Lambrecht et al., 2012; Sen et al., 2018), cognitive decline cannot fully account for performance deficits in emotion identification (Lambrecht et al., 2012). In addition, links between decreased emotional recognition and general cognitive decline are not strong (Ruffman and Sutcliffe, 2020). Thus, general cognitive decline in OA might not be the only reason that leads to decreased ability when recognising emotions.

Another contributing factor may be age-related changes in auditory perception. Older adults show altered processing of specific acoustic cues (Lima et al., 2014) and reduced sensitivity to temporal and spectral features critical for decoding emotional prosody and nonverbal vocalizations (Mitchell and Kingston, 2014). Although some studies show that age-related hearing loss per se does not fully account for emotion recognition deficits (Christensen et al., 2019), diminished precision in processing acoustic cues may still make it more difficult for OA to detect subtle characteristics. Importantly, changes in auditory processing might occur in individuals without clinically diagnosed hearing loss and thus may not be captured by self-report measures. Even though none of our participants reported impaired hearing, we did not conduct formal audiometric testing. Therefore, subtle age-related decreases in hearing sensitivity cannot be fully ruled out and may have influenced recognition ability in the present study.

A less frequently discussed explanation concerns motivational and task-design factors. It has been proposed that regarding task-design, laboratory emotion-recognition tasks might lack social meaning for OA, who might prioritize emotionally relevant interactions (Hamilton et al., 2022; Isaacowitz and Stanley, 2011). For example, younger and older women showed comparable empathic accuracy when recalling personal experiences that were highly relevant to them (Wieck and Kunzmann, 2015). In addition, in some studies age differences disappeared when a task-design was used in which participants were told that they must justify their judgments (Hess et al., 2009; Stanley and Isaacowitz, 2015). There is also evidence, that OA perform better when decoding emotions of familiar partners (Stanley and Isaacowitz, 2015). Thus, one could argue that due to a lack in motivation OA may allocate fewer cognitive resources than YA when interpreting laughter produced by unfamiliar individuals in laboratory settings. However, evidence that presenting stimuli in a more dynamic or contextually rich manner eliminates age effects in performance is mixed (Isaacowitz et al., 2017). Even when emotional expressions are more genuine, spontaneous, and ecologically valid, older adults generally perform worse than younger adults (Stanley and Isaacowitz, 2015; Wieck and Kunzmann, 2015). Thus, it remains unclear whether using a different paradigm would have reduced or eliminated age effects in emotion recognition.

A limitation of the present study concerns the age of the individuals producing the laughter stimuli. All laughter samples were produced by young adults, resulting in a potential speaker–listener age mismatch for the older participant group. Although own-age advantages are documented in face processing, it remains unclear if such effects also exist for decoding of emotional speech prosody. While research on face processing suggests that individuals show attentional and neural biases toward own-age faces (Ebner et al., 2011, 2013; He et al., 2011), evidence for a corresponding own-age advantage in emotion recognition is limited and mixed. Some studies even indicate that emotional expressions in younger faces are generally recognized more accurately than those in older faces, irrespective of the age of the observer (Ebner et al., 2011), or that age-related declines in recognition performance may be more pronounced for younger target faces (Lamont et al., 2005). Moreover, own-age biases appear to be more robust in face recognition memory than in emotion decoding and may depend on differential exposure to age groups (Anastasi and Rhodes, 2005; Harrison and Hole, 2009), with some studies finding such biases only in younger but not older adults (Denkinger and Kinn, 2018). Taken together, these findings suggest that although age-of-speaker effects cannot be ruled out, there is currently little evidence that individuals are generally more accurate in decoding emotional expressions from their own age group. Nevertheless, it remains possible that the observed age-related differences partly reflect reduced sensitivity of older adults to emotional cues expressed by younger speakers, rather than a general decline in laughter recognition per se. Future research should include laughter produced by both younger and older individuals to disentangle effects of listener age from potential age-of-poser influences in emotion recognition.

In summary, our findings demonstrate a robust age-related decline in laughter recognition across laughter types. While this decline may partly reflect age-related changes in cognitive resources, auditory processing, and motivational factors, existing evidence suggests that no single account fully explains older adults’ reduced performance, and age differences would likely persist even under more ecologically valid conditions.

### Pronounced age effects in schadenfreude classification

The difference in classification rates between younger and older adults was most pronounced for Schadenfreude laughter (differences in mean classification rates of YA vs. OA; Schadenfreude: 17%, Joy 4%, Tickling 2%). Evidence for the Positivity Effect, defined as the tendency of older adults to interpret social situations more positively than younger adults, has traditionally been based on studies which found pronounced age effects for so-called negative emotions, such as anger, sadness, and fear (Hayes et al., 2020; Mill et al., 2009; Nasrollahi et al., 2022; Ruffman et al., 2008). These emotions are typically characterized as unpleasant states experienced by the sender and are associated with distress, discomfort, or aversion in the sender. However, in a previous study we have shown that the valence of Schadenfreude laughter actually differs for the sender and receiver: While the sender (i.e., the person who laughs) is perceived to be in a pleasant state during Schadenfreude laughter, the receiver (i.e., the person who listens to the laughter) experiences the laugher as being unfriendly towards them (Szameitat et al., 2022). The present findings therefore extend previous work on the Positivity Effect by demonstrating that older adults interpret social cues more positively even when the negative quality of the emotion primarily affects the listener rather than the sender.

While direct evidence on age differences in schadenfreude laughter perception is missing, our results align with studies investigating the ability of OA and YA to differentiate literal statements vs. nonliteral statements such as sarcasm and teasing (Phillips et al., 2015; Pomareda et al., 2019; Rothermich et al., 2021). While there are many forms of nonliteral statements, in the present context it is of interest to focus on sarcasm, because it potentially shares similarities with schadenfreude laughter. Sarcastic statements are positively worded statements that have a negative or criticizing intention (Rothermich et al., 2021). Similarly, in Schadenfreude laughter, i.e., laughing at another’s misfortune, the usually positive signal of laughter has a negative intention. In other words, sarcasm and Schadenfreude both overlay a contradictory, “nonliteral meaning” which is negative in nature. Joy and Tickling laughter, on the other hand, can be considered to be literal forms of laughter, because the usually positive signal of laughter is coupled with a genuinely positive intention.

Previous studies on age effects in interpreting literal versus nonliteral statements found that, when viewing short video dialogues and judging speakers’ intentions, older adults were less accurate than younger adults in interpreting non-literal statements compared to literal statements (Phillips et al., 2015; Pomareda et al., 2019; Rothermich et al., 2021). More specifically, unlike younger adults older adults rated non-literal sarcastic statements as friendlier than literally negative statements (Rothermich et al., 2021) indicating that older adults may have difficulty identifying non-literal intent. This pattern corresponds with our observation that Schadenfreude laughter, which might also carry a non-literal meaning for the listener, was classified less accurately by older adults than by younger adults.

A further reason why age effects were more pronounced for Schadenfreude laughter may be that it represents a socially more complex form of laughter compared to more primary forms, such as tickling laughter (Kamiloğlu et al., 2024) and therefore requires greater cognitive effort and resources, which are known to decline with age (Zaninotto et al., 2018). Another possibility might be that OA have an increased Truth Bias (O’Connor et al., 2019) which suggests that people tend to assume honesty by default (Levine, 2014). This bias may make older adults less likely to suspect malicious intent in an originally affiliative signal leading them to misinterpret schadenfreude laughter.

In addition, the context of the stimulus creation might also have influenced schadenfreude recognition. Prior to the experiment, all participants were asked if they knew what the terms Schadenfreude, Joy, and Tickling mean and were asked for example scenarios. As all OA could give a valid explanation for all three terms it is unlikely that OA were less familiar with the term Schadenfreude than YA. However, the material that was used to provoke schadenfreude laughter, i.e., videoclips of pranks and others’ misfortunes taken from TV and the internet such as TikTok or Instagram, might have aligned more with younger adults’ social experiences, making schadenfreude more salient for YA. Noteworthy, we have shown that laughter recognition is significantly affected by culture (Szameitat and Szameitat, 2024). Schadenfreude extends beyond simple amusement at another person’s misfortune, as it reflects a socially complex emotional response influenced by social comparison, judgments of deservingness, envy, and moral evaluation (Smith et al., 2009). Therefore, the interpretation of schadenfreude-related laughter may partly depend on culturally shared norms and social experiences regarding when such laughter is perceived as appropriate or socially meaningful. Recent work further demonstrated that emotional meanings in German laughter can be recognised across cultures, while also showing advantages for listeners from culturally closer backgrounds (Szameitat and Szameitat, 2024). Thus, although all participants in the present study were German speakers and familiar with the concept of Schadenfreude, intergenerational and culturally shaped differences in social experiences may still have contributed to the pronounced age effects observed for schadenfreude laughter. These factors may therefore have influenced how readily schadenfreude laughter was recognized across age groups. Notably, the differences between age groups were less marked for tickling and joyful laughter, indicating these types may rely more on universal or less socially nuanced acoustic features. Tickling laughter, in particular, was recognized with relatively comparable accuracy across both groups (difference in classification rate: 2%), likely due to its unique acoustic characteristics (Kamiloğlu et al., 2024).

Taken together, the increased age-related decline in schadenfreude classification might be caused by greater social, cognitive, and acoustic complexity of this laughter type. Our results indicate that schadenfreude laughter might place higher cognitive demands on older adults than more basic forms of laughter such as joyful and tickling laughter. In addition, cultural differences in exposure to schadenfreude-related contexts, such as the prevalence of prank-based online media among younger adults, may further increase these age effects in recognition.

### Dominance perception

While there was no overall main effect of age on dominance ratings, we found that the effect of age was significantly different for the different laughter types (significant interactions between Age and Laughter Type). When the dominance ratings for YA and OA were compared directly, separate for each laughter type, OA tended to rate Schadenfreude laughter as less dominant than YA (*p* = 0.066), while OA and YA did not differ in the dominance ratings of Joy or Tickling. We take this pattern of significant interactions and the near-significant age effect in Schadenfreude as evidence that OA rated specifically Schadenfreude as less dominant than YA.

Regarding the dominance dimension, there is only limited evidence of age-related effects on the perception of vocal signals. In line with the current findings, von der Rosenthal- Pütten et al. (2019), who examined how younger and older adults interpreted communications produced by virtual agents, found that older adults judged the virtual agent to be more submissive, i.e., less dominant, than did younger adults. In addition, Fairfield et al. (2017), who investigated the perception of arousal, valence, and dominance in Italian words, reported that older adults assigned lower dominance ratings to words that younger adults had rated as moderately dominant. Assuming that dominance in social interactions is perceived as negative, these and our findings align with the positivity effect, i.e., that OA perceive negative stimuli (such as dominant stimuli or situations) as less negative than YA. However, McGettigan and Lavan (2023), who investigated the influence of listener age on the perception of various personality traits, found no differences between younger and older participants when judging dominance in audio recordings of sustained vowels. Therefore, while our findings and some prior research suggest that older adults might perceive vocal signals as less dominant as compared to younger adults, evidence is somewhat ambiguous and further studies are needed.

On a more general note, the current findings replicate and confirm our previous studies showing that Schadenfreude laughter is perceived as highly dominant in spontaneous natural laughter (Szameitat et al., 2022) as well as laughter produced by actors (Szameitat et al., 2009). While OA rated Schadenfreude as less dominant than YA, they still rated it as highly dominant. Therefore, we conclude that on a broader level, the perceived dominance patterns of joy, schadenfreude and tickling laughter are overall comparable between YA and OA.

## Conclusion

Overall, our findings suggest that while basic social cues in laughter remain accessible in older age, the decoding of socially more nuanced emotional signals becomes more challenging. Recognizing age-related changes in decoding of emotional nonverbal expressions can help the development of interventions, social programs, or communication strategies aimed at supporting older adults’ social engagement to maintaining strong interpersonal relationships.

## Data Availability

The original contributions presented in the study are publicly available. This data can be found here: Figshare, https://doi.org/10.17633/rd.brunel.32521458.
